# Acrolein Induces Changes in Cell Membrane and Cytosol Proteins of Erythrocytes

**DOI:** 10.3390/molecules29112519

**Published:** 2024-05-27

**Authors:** Michal Kopera, Krzysztof Gwozdzinski, Anna Pieniazek

**Affiliations:** 1 Doctoral School of Exact and Natural Sciences, University of Lodz, 90-236 Lodz, Poland; michal.kopera@edu.uni.lodz.pl; 2 Department of Oncobiology and Epigenetics, Faculty of Biology and Environmental Protection, University of Lodz, 90-236 Lodz, Poland; krzysztof.gwozdzinski@biol.uni.lodz.pl

**Keywords:** acrolein, erythrocytes, lipid membrane fluidity, thiol group, amino group

## Abstract

High concentrations of acrolein (2-propenal) are found in polluted air and cigarette smoke, and may also be generated endogenously. Acrolein is also associated with the induction and progression of many diseases. The high reactivity of acrolein towards the thiol and amino groups of amino acids may cause damage to cell proteins. Acrolein may be responsible for the induction of oxidative stress in cells. We hypothesized that acrolein may contribute to the protein damage in erythrocytes, leading to the disruption of the structure of cell membranes. The lipid membrane fluidity, membrane cytoskeleton, and osmotic fragility were measured for erythrocytes incubated with acrolein for 24 h. The levels of thiol, amino, and carbonyl groups were determined in cell membrane and cytosol proteins. The level of non-enzymatic antioxidant potential (NEAC) and TBARS was also measured. The obtained research results showed that the exposure of erythrocytes to acrolein causes changes in the cell membrane and cytosol proteins. Acrolein stiffens the cell membrane of erythrocytes and increases their osmotic sensitivity. Moreover, it has been shown that erythrocytes treated with acrolein significantly reduce the non-enzymatic antioxidant potential of the cytosol compared to the control.

## 1. Introduction

Acrolein (2-propenal) is a ubiquitous alkene, and an environmental contaminant [1]. This compound is found in large amounts in cigarette smoke [2,3]. However, this is not the only source of acrolein to which humans are exposed. Acrolein can also be generated endogenously as a metabolic product of the decomposition of polyamines and oxidation of threonine, as well as during lipid oxidation or the breakdown of anticancer drugs [4]. It has been reported that acrolein is associated with the induction and progression of many diseases [5]. These include, among others: rheumatoid arthritis, chronic obstructive pulmonary disease, acute alcoholic hepatitis, diabetes mellitus, Alzheimer’s disease, stroke, cancer diseases, chronic renal failure, and cardiovascular diseases [2,5]. The high reactivity of this molecule can lead to the formation of adducts with nuclear factors, proteases, and other proteins and disrupt the function of these biomolecules [4].

Cysteine thiol groups are most sensitive to reactions with acrolein. Cysteine thiol groups are highly susceptible to reacting with acrolein. The thiol groups of amino acids can react with acrolein through a nucleophilic addition process, resulting in the formation of mono- and dicysteine-acrolein adducts (known as the Michael addition reaction) [6]. Although the formation of adducts reduces the cytotoxicity of acrolein, they are still toxic to cells. Studies carried out on cells have shown that both acrolein and the resulting acrolein-cysteine conjugates have strong cytotoxic properties, and induce oxidative stress and apoptosis in cells [3,6]. However, the authors of this work showed that the formation of dicysteine-acrolein adducts reduces the cytotoxic effect in cells. The reaction of acrolein with the imidazole group of histidine or the amino group of lysine results in Michael addition products or Schiff base cross-links [7,8]. The reaction mechanism described above also applies to the binding of acrolein to the purine and pyrimidine bases of nucleic acids. It was found that acrolein forms adducts with guanine, adenine, and cytosine [7]. This may result in DNA damage generated by acrolein by forming DNA adducts, resulting in DNA cross-linking. Moreover, it has been shown that DNA−peptide/protein cross-link formation also occurs and influences DNA replication [9].

Another mechanism of action involves the generation of oxidative stress in cells in the presence of acrolein [10,11]. It has been shown that acrolein itself can cause oxidative damage, leading to the disruption of cell membranes and damage to DNA and mitochondria [12].

In cells exposed to acrolein, the level of reactive oxygen species (ROS) increases [6,13]. The high reactivity of acrolein towards thiol groups contributes to the reduction of the intracellular level of reduced glutathione. Moreover, a decrease in the activity of superoxide dismutase activity and glutathione peroxidase in ARPE-19 cells treated with acrolein was also observed [13]. Since acrolein is an aldehyde that is very reactive towards thiol and amino groups, we hypothesized that it may contribute to the formation of protein damage in erythrocytes, thus leading to a disruption of the proper structure of cell membranes.

The overall objective of the present study was to explore the role of acrolein in protein modification and the assessment of changes occurring in erythrocyte cell membranes exposed to its action. Particularly, we examined the modifications of proteins in hemolysate and plasma membrane proteins as well as the erythrocyte osmotic fragility and lipid membrane fluidity. Additionally, we assessed the level of non-enzymatic antioxidant potential and the concentration of lipid peroxidation products in the hemolysate of erythrocytes treated with acrolein. The obtained data are consistent with the hypothesis that acrolein induces cell-protein damage, causing at the same time changes in the fluidity of membrane lipids, an increase in carbonyl compounds, and a reduction in the non-enzymatic antioxidant potential of erythrocytes. This also confirms the reports of other researchers regarding the involvement of acrolein in the induction of oxidative stress.

## 2. Results

**Figure 1:**
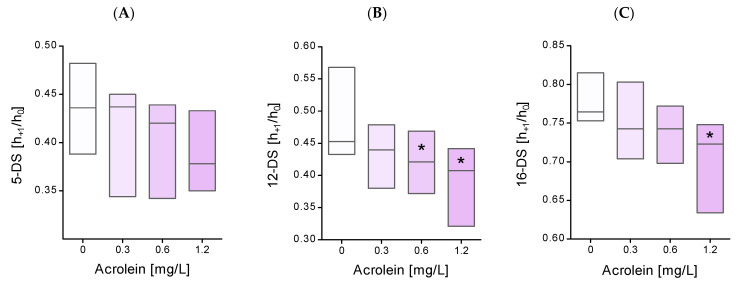
The h_+1_/h_0_ ratio of (**A**) 5-, (**B**) 12-, and (**C**) 16-doxyl-stearic acid incorporated into the membranes of whole erythrocytes after their incubation with acrolein. Data were presented as mean with a box plot of minimum and maximum values, n = 8, * *p* < 0.05—ACR (0.6 mg/L) and ACR (1.2 mg/L) versus control.

In the presented studies, the RBCs were incubated with acrolein at final concentrations of 0.3 mg/L (5.36 µM), 0.6 mg/L (10.71 µM), and 1.2 mg/L (21.43 µM) for 24 h at 37 °C. Immediately after the incubation of erythrocytes with acrolein, the lipid fluidity of the outer monolayer of the cell membrane was also examined. The lipid fluidity was assessed at the depth of the 5th, 12th, and 16th carbon atoms of the hydrocarbon chain of fatty acids. The obtained results showed no changes in lipid fluidity in the subsurface area of the cell membranes of erythrocytes incubated with acrolein (Figure 1A). Changes in the mobility of 12-DS and 16-DS spin labels were observed at the hydrophobic core of the erythrocyte cell membrane exposed to acrolein. At the depth of the 12th carbon atom, significant differences in membrane lipid fluidity were observed in erythrocytes incubated with acrolein at concentrations of 0.6 and 1.2 mg/L compared to the control cells (Figure 1B). In turn, at the depth of the 16th carbon atom, significant differences in the fluidity of membrane lipids were observed only in erythrocytes treated with acrolein at a concentration of 1.2 mg/L and not in the control (Figure 1C).

**Figure 2:**
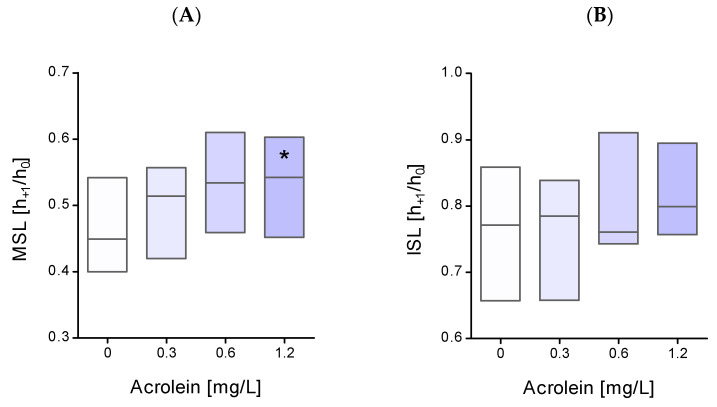
The h_+1_/h_0_ ratio of (**A**) MSL and (**B**) ISL attached to erythrocyte plasma membrane proteins after incubation of whole erythrocytes with acrolein. Data were presented as mean with a box plot of minimum and maximum values, n = 8, * *p* < 0.05—ACR (1.2 mg/L) versus control.

The conformational state of erythrocyte membrane proteins incubated with acrolein was also analyzed using spin labels (MSL and ISL). The obtained research results showed a significant change in the conformational state of erythrocyte membrane proteins measured using MSL after their incubation with acrolein at the highest concentration (1.2 mg/L) (Figure 2A). In the case of ISL, an increase in the h_+1_/h_0_ ratio of the label attached to erythrocyte membrane proteins for higher acrolein concentrations was observed, but the results were not statistically significant (Figure 2B).

**Figure 3:**
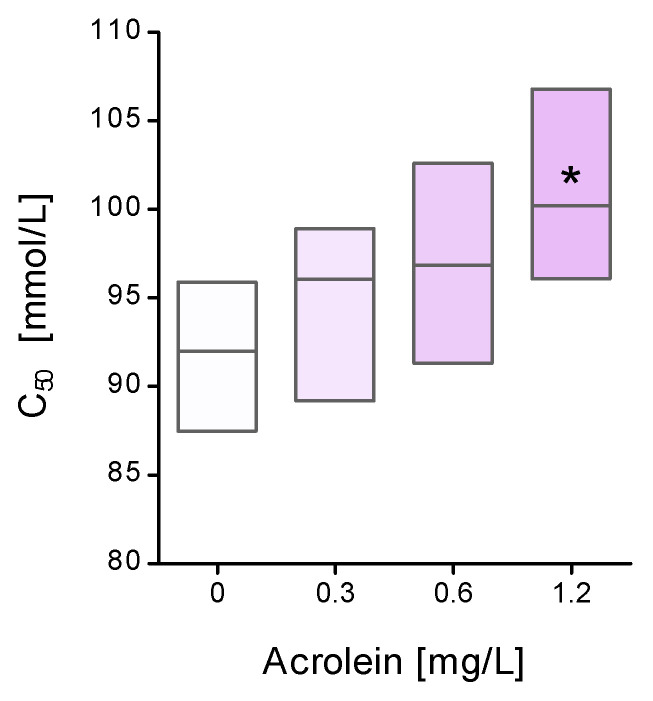
Osmotic fragility of the RBCs after incubation with acrolein (C_50_—NaCl concentration at which 50% of RBCs undergo hemolysis). Data were presented as mean with a box plot of minimum and maximum values, n = 7, * *p* < 0.05—ACR (1.2 mg/L) versus control.

Immediately after the end of incubation, the osmotic fragility of RBCs was assessed by determining the NaCl concentration at which half of the cells undergo hemolysis. Based on the conducted research, it was found that RBCs treated with acrolein at the highest concentration of 1.2 mg/L were significantly more sensitive to hemolysis than the control cells, as shown in Figure 3 and (Supplementary Materials). On the other hand, the remaining two concentrations of acrolein, 0.3 and 0.6 mg/L, caused only a slight increase in the osmotic sensitivity of the erythrocytes.

**Figure 4:**
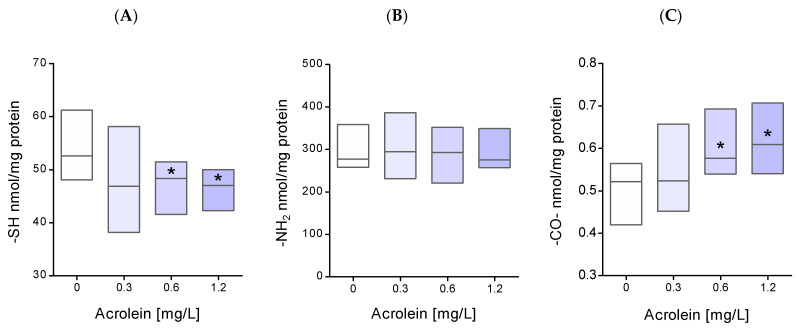
The level of (**A**) thiol, (**B**) amino, and (**C**) carbonyl groups in erythrocyte plasma membrane proteins after incubation of whole erythrocytes with acrolein. Data were presented as mean with a box plot of minimum and maximum values, n = 9, * *p* < 0.05—ACR (0.6 mg/L) and ACR (1.2 mg/L) versus control.

Functional groups of peptides and proteins are highly sensitive to reactions with acrolein. For this reason, the level of thiol, amino, and carbonyl groups in the membrane proteins of erythrocytes exposed to acrolein was analyzed. The conducted research showed a significant decrease in the level of thiol groups in the membrane proteins of erythrocytes exposed to acrolein at a concentration of 0.6 and 1.2 mg/L (Figure 4A). Acrolein at a concentration of 0.3 mg/L caused a slight decrease in the level of thiol groups in membrane proteins. None of the applied concentrations of acrolein affected changes in the level of amino groups in erythrocyte membrane proteins (Figure 4B). Furthermore, a significant increase in the level of carbonyl groups was observed in the membrane proteins of erythrocytes treated with acrolein at concentrations of 0.6 and 1.2 mg/L, compared to the values obtained for the control (Figure 4C).

**Figure 5:**
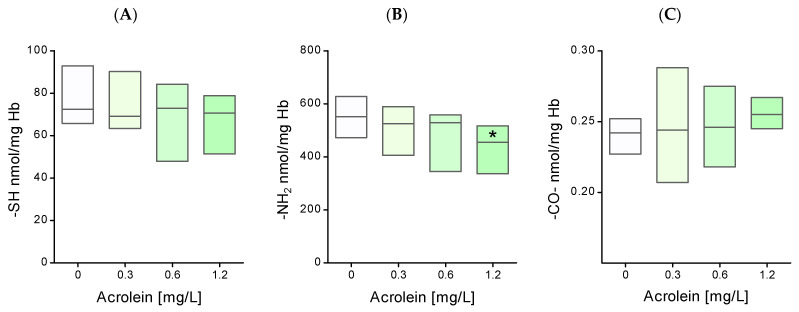
The level of (**A**) thiol, (**B**) amino, and (**C**) carbonyl groups of hemolysate proteins of erythrocytes incubated with acrolein. Data were presented as mean with a box plot of minimum and maximum values, n = 9, * *p* < 0.05—ACR (1.2 mg/L) versus control.

An analogous study of the levels of thiol, amine, and carbonyl groups was carried out in the hemolysate of acrolein-treated erythrocytes. The results showed a slight reduction in the level of thiol groups in the hemolysate proteins of erythrocytes incubated with acrolein compared with the values obtained for the control (Figure 5A). On the other hand, acrolein at a concentration of 1.2 mg/L contributed to a significant decrease in the level of amino groups in the proteins inside the erythrocytes compared to the control (Figure 5B). No significant differences in the level of carbonyl groups were observed in the cytoplasmic proteins of erythrocytes exposed to acrolein compared to those of the control erythrocytes (Figure 5C).

**Figure 6:**
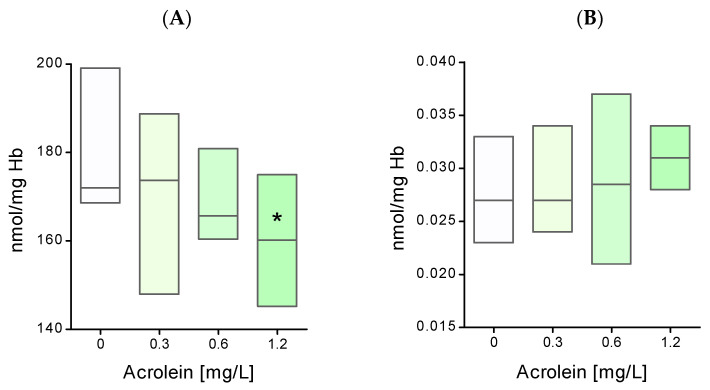
(**A**) The total non-enzymatic antioxidant potential and (**B**) the concentration of thiobarbituric acid-reactive peroxidation products (TBARS) in hemolysate of erythrocytes incubated with acrolein. Data were presented as mean with a box plot of minimum and maximum values, n = 9, * *p* < 0.05—ACR (1.2 mg/L) versus control.

The presence of acrolein in biological systems may generate oxidative stress. Therefore, the level of total non-enzymatic antioxidant potential and the concentration of thiobarbituric acid-reactive peroxidation products (TBARS) were determined in the hemolysate of erythrocytes incubated with acrolein. The obtained research results showed that acrolein at a concentration of 1.2 mg/L causes a significant decrease in the total non-enzymatic antioxidant potential compared to the control (Figure 6A). However, TBARS levels in the hemolysate did not change significantly compared to the control after the incubation of erythrocytes with acrolein (Figure 6B).

## 3. Discussion

Initially, the acrolein concentrations selected for testing did not exceed 0.1 mg/L, which corresponded to the values observed in patients with chronic kidney disease [14]. However, after performing several tests examining osmotic fragility and conformational changes in erythrocyte membrane proteins, we did not observe any significant differences compared to the control. Moreover, when selecting acrolein concentrations for testing, we did not want to exceed the IC50 doses for various cells found in the literature. It has been shown that 24 hour incubation of HUVECs with acrolein at a concentration of 50 µmol/L causes a decrease in their survival by approximately 40% [15]. Similar results were obtained for the HepG2 cell line incubated with acrolein for 24 hours [16]. On the other hand, studies conducted on Caco-2 and GES-1 cells showed that after 48 h of incubation at a concentration of 20 µmol/L, less than 50% of the cells survived [17]. These studies show that the cytotoxicity of acrolein depends on the type of cells exposed to it and the exposure time.

The first shield against the action of toxins on cells is the cell membrane. Our studies showed an increase and changes in the properties of the erythrocyte plasma membrane. The membrane fluidity depends on various factors such as the type of lipids present, the number of unsaturated bonds in fatty acids, and the ratio of proteins to lipids in the lipid bilayer of the membrane, as well as lipid–protein interactions.

Our experiment, using 12-DS and 16-DS fatty acid spin probes, revealed that the exposure of erythrocytes to acrolein results in a decrease in lipid fluidity in the hydrophobic region of the lipid monolayer. This decrease in lipid fluidity can be attributed to the changes in protein–lipid interactions. It appears that proteins are more susceptible to the influence of acrolein. We previously mentioned that acrolein reacts with cysteine, but it can also form heterocyclic adducts with lysine residues. Thus, the interaction of lipids with modified proteins may lead to a reduction in membrane fluidity. However, changes occurring at the level of membrane lipids are certainly not the only factor responsible for the disruption of the erythrocyte cell membrane. Our study showed conformational changes in the membrane cytoskeleton proteins measured using the MSL spin-label. This spin-label binds covalently to the thiol groups of cytoskeletal proteins, especially to the spectrin–actin complex [18]. In these studies, we observed an increase in the mobility of membrane cytoskeletal proteins, mainly in the spectrin-actin complex. These results explain the higher osmotic sensitivity of erythrocytes treated with acrolein. The obtained results are consistent with those of other reports, where under the influence of acrolein, a significant increase in the hemolysis of erythrocytes and their morphological changes towards the formation of echinocytes was observed [19,20]. Our studies have shown that the lipid membrane undergoes significant stiffening in the hydrophobic parts of the membrane. An increase in membrane stiffness was observed in echinocytes [21]. Other work has shown that acrolein increases the formation of ceramides and the externalization of phosphatidylserine in erythrocyte cell membranes. The authors of these studies suggest that this may be the cause of morphological changes in these cells and, consequently, the induction of eryptosis [19]. Moreover, we also observed a decrease in the number of thiol groups and an increase in carbonyl group levels in plasma membrane proteins after erythrocyte incubation with acrolein. The decrease in the level of thiol groups may result, firstly, from the high reactivity of acrolein to thiols, and secondly, from the oxidation of thiol groups in proteins by reactive oxygen species. The high reactivity of acrolein towards thiol groups can be confirmed by a significant decrease in the level of reduced glutathione observed by several research teams [13,20,22,23].

The non-enzymatic antioxidant potential is related to the presence of all low-molecular-weight cellular reducers that can capture reactive oxygen species. The decrease in glutathione concentration may contribute to the reduction of the total non-enzymatic antioxidant potential of cells, which we observed in erythrocytes incubated with acrolein. On the other hand, a decrease in the non-enzymatic antioxidant potential of cells may result from an increase in the level of reactive oxygen species. The ability of acrolein to generate reactive oxygen species in cells has been experimentally confirmed using several cell models treated with these toxins in original studies [6,24,25]. Moreover, the work of O’Toole et al. [24] showed that exposure to 25 μM acrolein increased the ROS in macrophages to a similar extent as in cells treated with 100 μM H_2_O_2_ described as a positive control. Some reports have shown that acrolein creates adducts with protein kinase C in the mitochondria which results in a decrease in its activity and intra-mitochondrial signaling [26]. In this way, changes occur in the functioning of the respiratory chain in mitochondria and contribute to the increased production of reactive oxygen species.

An increase in the level of reactive oxygen species may result in the induction of oxidative stress, which manifests a decrease in the antioxidant potential of cells. In ARPE-19 cells treated with acrolein, a decrease in glutathione peroxidase and superoxide dismutase activity was observed [13]. Our studies have shown that acrolein contributes to the reduction of the total non-enzymatic antioxidant potential of erythrocytes. Moreover, in vitro studies demonstrated oxidative damage to proteins as a result of the action of acrolein in the form of an increase in the level of protein carbonyl groups [13,23]. Increased levels of carbonyl groups in the spleen, thymus, and polymorphonuclear leukocyte proteins were also observed in rats after acrolein administration [22]. The level of carbonyl groups in erythrocyte membrane proteins treated with acrolein also increased and these results are in agreement with those described above. However, our studies did not show significant changes in the concentration of carbonyl groups and thiol groups in the proteins of hemolysate isolated from erythrocytes treated with acrolein. One molecule of hemoglobin (2α2β chains) contains 6 Cys, 38 His, 44 Lys, and 4 N termini amino groups, which gives potentially 92 acrolein binding sites [27]. Considering the small number of cysteine residues, changes in the level of thiol groups in hemolysate proteins may be insignificant in this case. It appears that the first target of acrolein’s action after entering erythrocytes is cytoskeletal proteins located on the inner monolayer of the membrane. This may also be demonstrated by the significant decrease in the level of thiol groups we observed in the membrane proteins of erythrocytes incubated with acrolein.

Taking into account the fact that acrolein reacts with the amino groups of proteins, we expected a reduction in the level of these groups in hemolysate proteins and erythrocyte membrane proteins. We observed a significant decrease in the level of amino groups only in hemolysate proteins after the incubation of erythrocytes with the highest concentration of acrolein (1.2 μg/L). These results confirm previous reports regarding the modification of hemoglobin treated with acrolein [8]. The authors of this work identified a total of 26 different sites and types of modified peptides. The N-propene adducts were detected in three lysines and three histidines, and the N-propanol adducts were found in one cysteine, six histidine, and 13 lysine residues [8]. Similar research results regarding the modification of lysine and histidine in hemoglobin under the influence of acrolein were also obtained by Lasse et al. [27]. Furthermore, mass spectra obtained by this team indicate that the degree of change in hemoglobin depends on the acrolein concentration.

The consequence of oxidative stress is the oxidation of proteins and lipids in cells. Therefore, as expected, a significant increase in TBARS levels was observed in HepG2 cells incubated with acrolein [23]. However, the authors of this study used acrolein in doses 4–6 times higher than those used in our studies. Similarly, an increase in TBARS levels was also found in the spleen, thymus, and polymorphonuclear leukocytes in rats treated with oral doses of acrolein for 30 days [22]. In our studies, we observed a slight increase in the levels of thiobarbituric acid-reactive products after the treatment of erythrocytes with acrolein. However, we demonstrated an increase in the level of carbonyl compounds in erythrocyte cell membranes, which indicates the initiation of oxidative stress by acrolein.

In summary, our studies have demonstrated that acrolein has a significant impact on cell membrane and hemolysate proteins. Specifically, acrolein alters cell membrane fluidity, membrane cytoskeleton proteins, and increases cell sensitivity to hemolysis. We have observed a decrease in the concentration of free thiol and amino groups, as well as an increase in the number of carbonyl groups due to exposure to acrolein in cell membrane proteins. Additionally, acrolein results in a significant reduction in the non-enzymatic antioxidant potential within erythrocytes. The extent of these changes in erythrocytes is dose-dependent. Notably, our findings indicate that the decrease in membrane fluidity is attributed to a change in protein–lipid interactions rather than lipid peroxidation.

## 4. Material and Methods

### 4.1. Chemicals

The following chemicals were purchased from Sigma Chemical Co. (St. Louis, MO, USA): 4-maleimide-2,2,6,6,-tetramethylpiperidine-1-oxyl (MSL), 4-iodoacetamide-2,2,6,6,-tetramethylpiperidine-1-oxyl (ISL), 5-doxyl-stearic acid (5-DS), 12-doxyl-stearic acid (12-DS), 16-doxylstearic acid (16-DS), (5,5′-dithiobis (2-nitrobenzoic acid) (DTNB), 4,4-dithiodipyridine, 2,4,6-trinitrobenzene sulfonic acid (TNBS), 2,4-dinitrophenylhydrazine (DNPH), and 2,4,6-tripyridyl-S-triazine (TPTZ).

Acrolein for research was synthesized based on the method of Adkins and Hartun [28] with modifications by Maksorov and Andrianow [29] by heating glycerol with potassium sulfates (KHSO_4_ and K_2_SO_4_). Pure UV spectra of acrolein were measured on a Carry spectrophotometer in hexane in the wavelength range 200–400 nm (Supplementary Materials). UV spectra (λ_max_ 210 nm and 340 nm) of the obtained colorless liquid with a characteristic odor were identical to the authentic sample presented in the NIST Chemistry webbook [30].

Unless otherwise indicated, all other chemicals were purchased from POCH S.A. (Gliwice, Poland).

### 4.2. Sample Preparation

The experiments were performed on erythrocytes isolated from the human blood buffy coat obtained from the Blood Bank in Lodz. (Franciszkanska 17/25, 91-433 Łodz, Poland). Erythrocytes are a convenient research model for analyzing cellular membrane changes. Their lack of a nucleus and mitochondria simplifies result interpretation by excluding acrolein’s interactions with DNA or mitochondrial membrane proteins.

Erythrocytes were isolated from human blood buffy coat by centrifugation and washing three times with PBS (10 mM phosphate buffered saline, pH 7.4). The isolated erythrocytes were then diluted in Ringer’s buffer to 50% hematocrit and incubated with acrolein concentrations (0.3 mg/L [5.36 µM], 0.6 mg/L [10.71 µM], and 1.2 mg/L [21.43 µM]) for 24 h at 37 °C. A stock solution of acrolein, used in the study, was prepared in PBS at a concentration of 10 mg/mL. The control samples contained a PBS solution. After incubation, erythrocytes were washed with Ringer’s buffer and used for further experiments.

Erythrocyte plasma membranes were isolated from erythrocytes using the method described by Dodge et al. [31]. The concentration of plasma membrane proteins was measured spectrophotometrically using the Folin–Ciocalteu reagent, with an absorption maximum of 750 nm [32].

The hemolysate was prepared by mixing erythrocytes with cold water in a ratio of 1:1.5. The samples were centrifuged to remove the erythrocyte membranes and then the hemoglobin (Hb) concentration in the hemolysate was determined spectrophotometrically as cyanmethemoglobin using Drabkin’s reagent at an absorption maximum of 540 nm [33].

### 4.3. Measurement of Lipid Membrane Fluidity

Erythrocyte lipid membrane fluidity was measured using the electron paramagnetic resonance (EPR) technique with three spin probes, derivatives of doxyl-stearic acids: 5-, 12-, and 16-DS. The tracers in 0.1 mM ethanol solution were added to the whole erythrocyte samples and incubated for 0.5 h at 4 °C. The EPR spectra of spin labels, incorporated with the erythrocyte membranes, were used to calculate the h_+1_/h_0_ parameter. EPR spectra were recorded using a Bruker ESP 300 E spectrometer (Rheinstetten, Germany, microwave frequency of 9.73 GHz). The instrumental settings were as follows: center field, 3480 G; scan range, 80 G; modulation frequency, 100 kHz; modulation amplitude, 1 G.

### 4.4. Measurement of the Conformational State of Plasma Membrane Proteins

The spin labels MSL (4-maleimide-2,2,6,6,-tetramethylpiperidine-1-oxyl) and ISL (4-iodoacetamide-2,2,6,6,-tetramethylpiperidine-1-oxyl) were used to study the conformational changes of plasma membrane proteins. Erythrocyte membrane proteins were labeled with alcohol solutions of MSL or ISL for 1 h at 4 °C. The alcohol concentration in the samples did not exceed 0.002%. To remove excess labels, the samples were washed 3 times with 5 mM phosphate buffer pH = 7.4. Using the obtained EPR spectra for the samples, the h_+1_/h_0_ parameter was calculated.

### 4.5. Osmotic Fragility Measurement

The osmotic fragility of red blood cells was measured using the method described by Morimoto by measuring the absorbance (540 nm) in the supernatants of erythrocyte samples incubated in solutions with different NaCl concentrations (0–155 mM) [34]. Based on the obtained results, the concentration of NaCl in which 50% of erythrocytes undergo hemolysis was calculated.

### 4.6. Measurement of Thiol-Group Content

The concentration of free thiol groups in membrane proteins was measured using the Ellman method [35]. During the reaction of Ellman’s reagent ((5,5′-dithiobis (2-nitrobenzoic acid); DTNB) with free thiol groups, the optically active 2-nitro-5-thiobenzoate (NTB) is formed. The absorbance of the formed NTB was measured at a wavelength of 412 nm.

The concentration of free thiol groups in the hemolysate was measured using 4-4′-dithiodipyridine [36]. When reacting with the free thiol groups, a colored product is formed: 2-thiopyridone, the concentration of which is measured spectrophotometrically at 324 nm.

For both methods, calibration curves were prepared for various concentrations of reduced glutathione (0–1 mM), based on which the concentration of thiol groups in proteins was calculated. The concentration of thiol groups was expressed as nmol/mg of membrane protein or mg of Hb.

### 4.7. Measurement of Amino-Group Content

The concentration of free amino groups in the hemolysate and membrane proteins was determined using the method described by Crowell et al. [37]. This method involves the reaction of 2,4,6-trinitrobenzene sulfonic acid (TNBS) with amino groups to form colored products, which are then measured spectrophotometrically at 335 nm. The concentration of free amino groups was read from a standard curve prepared for homocysteine solutions at concentrations ranging from 0 to 250 μM and expressed in nmol/mg of membrane protein or mg of Hb.

### 4.8. Measurement of Carbonyl-Group Content

The content of carbonyl groups in hemolysate and plasma membrane proteins was determined using the method described by Levine et al. [38]. The method uses the reaction of 2,4-dinitrophenylhydrazine (DNPH) with carbonyl groups from proteins, which is optically active at 370 nm, to form dinitrophenylhydrazone (DNP). The concentration of carbonyl groups was calculated using the molar absorption coefficient (22 mmol^−1^·cm^−1^) and expressed as nmol/mg of membrane protein or mg of Hb.

### 4.9. Measurement of Total Non-Enzymatic Antioxidant Capacity

The total non-enzymatic antioxidant capacity (NEAC) of the hemolysate was measured using the reduction of the ferric-2,4,6-tripyridyl-s-triazine [Fe(III)-TPTZ] complex to the [Fe(II)-TPTZ] complex, which is optically active at 593 nm, by cellular antioxidants [39]. Antioxidant levels were calculated from the calibration curve prepared for different concentrations of Trolox (0–1 mmol/L) and expressed as nmol Trolox equivalents per milligram of membrane protein or mg of Hb.

### 4.10. Measurement of Thiobarbituric-Acid-Reactive Substance Content

Lipid peroxidation in the hemolysate was assessed using the reaction of the end products of lipid oxidation with thiobarbituric acid at low pH (TBARS), resulting in the formation of colored products. The end product of the reaction was determined at 535 nm [40]. To calculate the concentration of TBARS, the millimolar absorption coefficient (156 mmol^−1^·cm^−1^) was used and the data were expressed as nmol/mg Hb.

### 4.11. Statistical Analysis

Statistical analysis of the data involves checking the normality of the distribution of the tested parameters using the Shapiro–Wilk test. Then, the homogeneity of variances was checked using Levene’s test. The results of the above tests allowed analysis by one-way ANOVA using a post hoc multiple Tukey’s comparisons test. Statistical significance was accepted at *p* < 0.05. The statistical analysis was performed using Statistica v. 13.3 (StatSoft Polska, Krakow, Poland). Data in the figures were presented as means with a box plot of minimum and maximum values.

## 5. Conclusions

Our studies have shown that erythrocytes are more resistant to the action of acrolein compared to nucleated cells such as HUVEC, HEPG-2 Caco-2, GEST-1, and possibly others. We demonstrated that acrolein primarily targets the cell membrane, using erythrocytes as an example. The cells’ sensitivity to oxidative damage may have increased due to a reduction in thiols and non-enzymatic antioxidant potential.

## Data Availability

Data can be made available after contacting the corresponding author.

## Supplementary Materials

The following supporting information can be downloaded at: https://www.mdpi.com/article/10.3390/molecules29112519/s1, Figure S1. UV spectra of acrolein in hexane. Figure S2. Hemolysis curves of erythrocytes after incubation with acrolein.
