# Supplementary material for: Acrolein Induces Changes in Cell Membrane and Cytosol Proteins of Erythrocytes
Source: Molecules. 2024 May 27;29(11):2519. doi: 10.3390/molecules29112519 (PMC11173626; doi:10.3390/molecules29112519)
Supplement: Supplementary file 1 [file molecules-29-02519-s001.zip › molecules-2983834-supplementary.pdf]

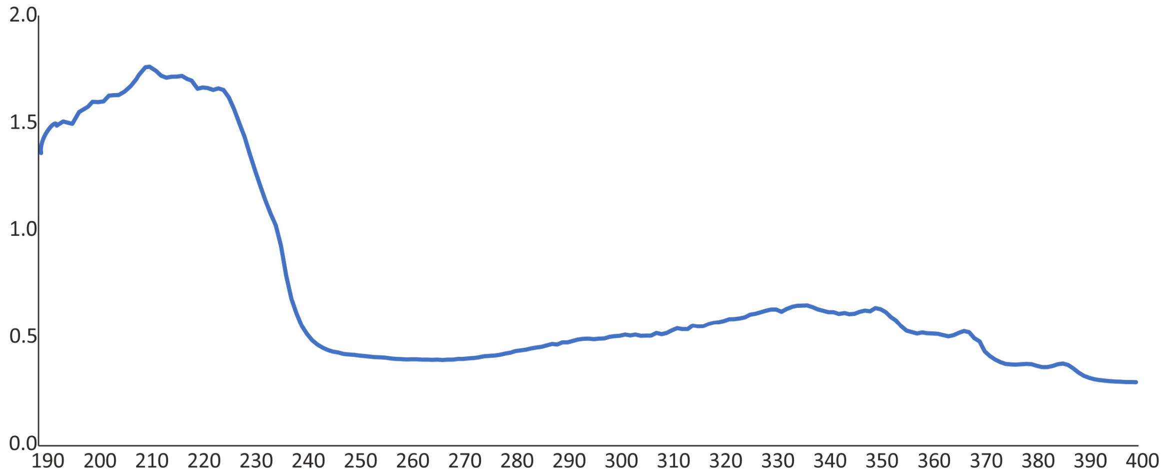

Figure S1. UV spectra of acrolein in hexane

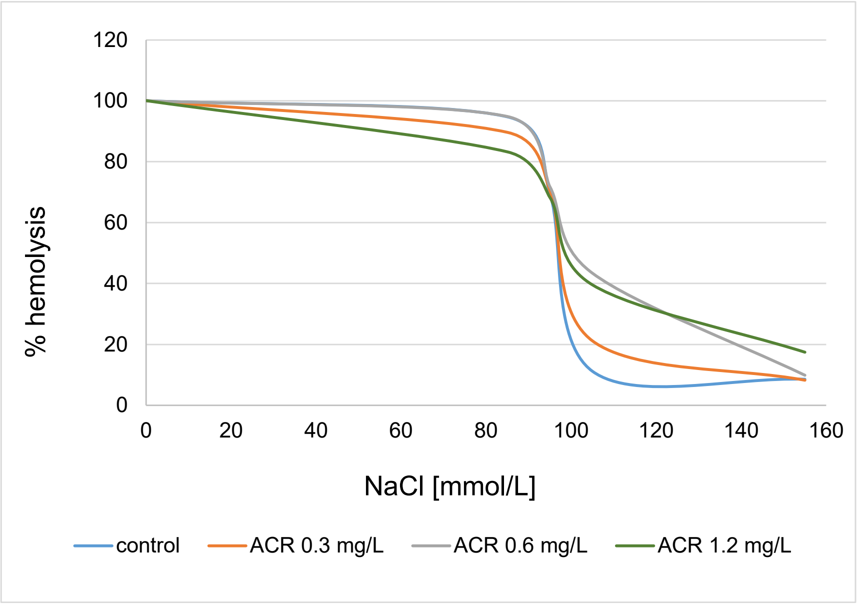

Figure S2. Hemolysis curves of erythrocytes after incubation with acrolein
